# Cells adapt to the epigenomic disruption caused by histone deacetylase inhibitors through a coordinated, chromatin-mediated transcriptional response

**DOI:** 10.1186/s13072-015-0021-9

**Published:** 2015-09-16

**Authors:** John A Halsall, Nil Turan, Maaike Wiersma, Bryan M Turner

**Affiliations:** Chromatin and Gene Expression Group, School of Cancer Sciences, College of Medical and Dental Sciences, University of Birmingham, Birmingham, B15 2TT UK

**Keywords:** Histone modification, Chromatin, Gene expression, Histone deacetylase inhibitors, Polycomb complex, Valproic acid, Suberoylanilide hydroxamic acid

## Abstract

**Background:**

The genome-wide hyperacetylation of chromatin caused by histone deacetylase inhibitors (HDACi) is surprisingly well tolerated by most eukaryotic cells. The homeostatic mechanisms that underlie this tolerance are unknown. Here we identify the transcriptional and epigenomic changes that constitute the earliest response of human lymphoblastoid cells to two HDACi, valproic acid and suberoylanilide hydroxamic acid (Vorinostat), both in widespread clinical use.

**Results:**

Dynamic changes in transcript levels over the first 2 h of exposure to HDACi were assayed on High Density microarrays. There was a consistent response to the two different inhibitors at several concentrations. Strikingly, components of all known lysine acetyltransferase (KAT) complexes were down-regulated, as were genes required for growth and maintenance of the lymphoid phenotype. Up-regulated gene clusters were enriched in regulators of transcription, development and phenotypic change. In untreated cells, HDACi-responsive genes, whether up- or down-regulated, were packaged in highly acetylated chromatin. This was essentially unaffected by HDACi. In contrast, HDACi induced a strong increase in H3K27me3 at transcription start sites, irrespective of their transcriptional response. Inhibition of the H3K27 methylating enzymes, EZH1/2, altered the transcriptional response to HDACi, confirming the functional significance of H3K27 methylation for specific genes.

**Conclusions:**

We propose that the observed transcriptional changes constitute an inbuilt adaptive response to HDACi that promotes cell survival by minimising protein hyperacetylation, slowing growth and re-balancing patterns of gene expression. The transcriptional response to HDACi is mediated by a precisely timed increase in H3K27me3 at transcription start sites. In contrast, histone acetylation, at least at the three lysine residues tested, seems to play no direct role. Instead, it may provide a stable chromatin environment that allows transcriptional change to be induced by other factors, possibly acetylated non-histone proteins.

**Electronic supplementary material:**

The online version of this article (doi:10.1186/s13072-015-0021-9) contains supplementary material, which is available to authorized users.

## Background

Levels of histone acetylation across the genome reflect a dynamic equilibrium between the activities of two enzyme families, lysine acetyltransferases (KATs) and histone deacetylases (HDACs) [1, 2]. HDAC activity can be suppressed by a variety of naturally occurring and synthetic compounds, resulting in a detectable increase in global histone acetylation after 10 min or less, and hyperacetylation of over 90 % of H4 molecules after just a few hours, indicating that most of the genome is acted on by KATs and HDACs ([3, 4] and references therein). This conclusion is consistent with studies on the genomic distribution of these enzymes [5–9].

Like other post-translational histone modifications, acetylation rarely acts in isolation, but as part of a combination of different modifications, usually along the histone N-terminal tails, that collectively regulate chromatin function [10–12]. They do this either by directly influencing chromatin structure, or by serving as docking sites for non-histone proteins which, in turn, exert functional change [13–15]. In view of this, generalisations about the functional roles of particular modifications are rarely appropriate or useful. However, there is a long-standing connection between relatively high levels of overall histone acetylation and transcriptional activity [16–18]. This association is supported by more recent epigenomic studies [19–24], and by experiments on the functional consequences of acetylation of specific histone lysines. For example, enhanced acetylation of H4 specifically at lysine 16 is a marker of the transcriptionally hyperactive male X chromosome in *D. melanogaster* [25, 26] and has recently been linked more generally to transcriptionally active genes [27]. H3K9 acetylation is consistently enhanced at gene promoter regions [28, 29], while H3K27 acetylation protects this residue from methylation by the Polycomb silencing Complex PRC2 and consequent long-term suppression of transcription [30, 31].

In view of this, it is puzzling that cells can tolerate so well the massive hyperacetylation of core histones, and other proteins, caused by histone deacetylase inhibitors (HDACi). Many cultured cell types, including non-transformed lines such as mouse embryonic stem cells, continue to grow, albeit slowly, in the presence of HDACi [32, 33] and whole organisms continue to function [34, 35]. Indeed, various HDACi have been in clinical use for many years. Valproic acid (VPA), a short-chain fatty acid, is an effective anti-epileptic and mood stabiliser [36], while VPA and chemically more complex HDACi such as hydroxamic acid derivatives and depsipeptide, have been tested against a variety of cancers [37–40]. It has been known for some time that cultured cells treated with HDACi do not undergo a global up-regulation of transcription. In fact, only a small proportion of genes significantly change expression, and up to half of these are down-regulated [41–45]. These findings raise fundamental questions regarding the relationship between histone acetylation and transcription, and about the mechanisms by which cells might protect their transcriptional programmes from the potentially disruptive effects of induced epigenetic change.

Attempts to define the processes through which HDACi influence cell function, are complicated by the fact that they usually inhibit several different members of the 18-strong HDAC family. The most commonly used HDACi, including short-chain fatty acids and hydroxamic acid derivatives, inhibit the class I and IIa enzymes, HDACs 1, 2, 3, 6 and 8, of which HDACs 1–3 are consistently chromatin associated and likely to be key players in regulation of gene expression [5]. These enzymes are catalytically active only when physically associated with specific partner proteins and four complexes have been isolated and characterised, namely CoRest, NuRD, Sin3 and NCoR/SMRT [46–48]. Class IIb and IV enzymes have little or no catalytic activity, while the NAD-dependent Class III enzymes (the Sirtuins, SIRT1-7) have a different catalytic mechanism and are unaffected by HDACi [49, 50]. Finally, each of the class I/IIa HDACs has multiple substrates, both histones and non-histone proteins, including various acetyltransferases and deacetylases [51–53].

Most previous work to explore cellular responses to HDACi has used treatment times of at least 4 h and often 24 h or longer, making it impossible to identify the key processes that underpin, and initiate, what is inevitably a complex and changing response. The experiments described here define the sequential transcriptional and histone modification changes that constitute the early response (within 2 h) of human cells to VPA and suberoylanilide hydroxamic acid (SAHA). The results reveal a coordinated transcriptional response that promotes cell survival by minimising protein hyperacetylation, slowing growth and re-balancing patterns of gene expression. Unexpectedly, the response involves a precisely timed increase in H3K27me3 at transcription start sites, but little or no increase in histone acetylation, whose role seems to be to provide a stable chromatin environment that allows transcription to be modified by other factors.

## Results

All experiments were carried out with human lymphoblastoid cell lines, derived from B-lymphocytes immortalized, but not fully transformed, by Epstein Barr Virus (EBV, [54]). To explore the earliest transcriptional responses to HDACi, we treated cells, in triplicate, with either sodium valproate or SAHA for 0, 30, 60 and 120 min. We tested three concentrations of each inhibitor, covering a 25-fold range. A progressive increase in histone acetylation was detectable by western blotting at all concentrations of both inhibitors (Additional file 1A). No change in cell cycle profile or the frequency of apoptotic cells was detectable within 120 min, but after 24 h both inhibitors slowed cell cycle progression and, at the highest concentrations tested, induced a small proportion (8–10 %) of apoptotic cells (Additional file 1B).

**Fig. 1:**
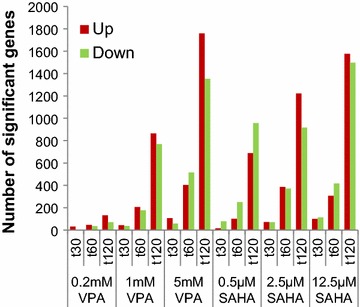
Concentration and time dependence of the transcriptional response to HDACi. Histogram showing the number of genes whose transcript levels are significantly increased (*red*) or decreased (*green*) at three time points and three concentrations of VPA or SAHA, as indicated (*t* test, *P* < 0.05, fold change >1.5).

To monitor changes in transcription, fluorescently-labelled cDNA from each sample was applied to a Nimblegen HD2 135K array, on slides each containing 12 such arrays. Thus, a time-course experiment (0, 30, 60, 120 min), in triplicate, could be accommodated on a single slide. We used *t* tests (*P* < 0.05, fold change >1.5) to identify genes whose transcript levels were significantly changed, relative to *t*_0_, at each time point. After 120 min at the highest concentration of each inhibitor, about 7 % of elements on the array showed significantly up- or down-regulated expression, in approximately equal numbers (Fig. 1).

### Defined gene populations show characteristic expression changes over time

**Fig. 2:**
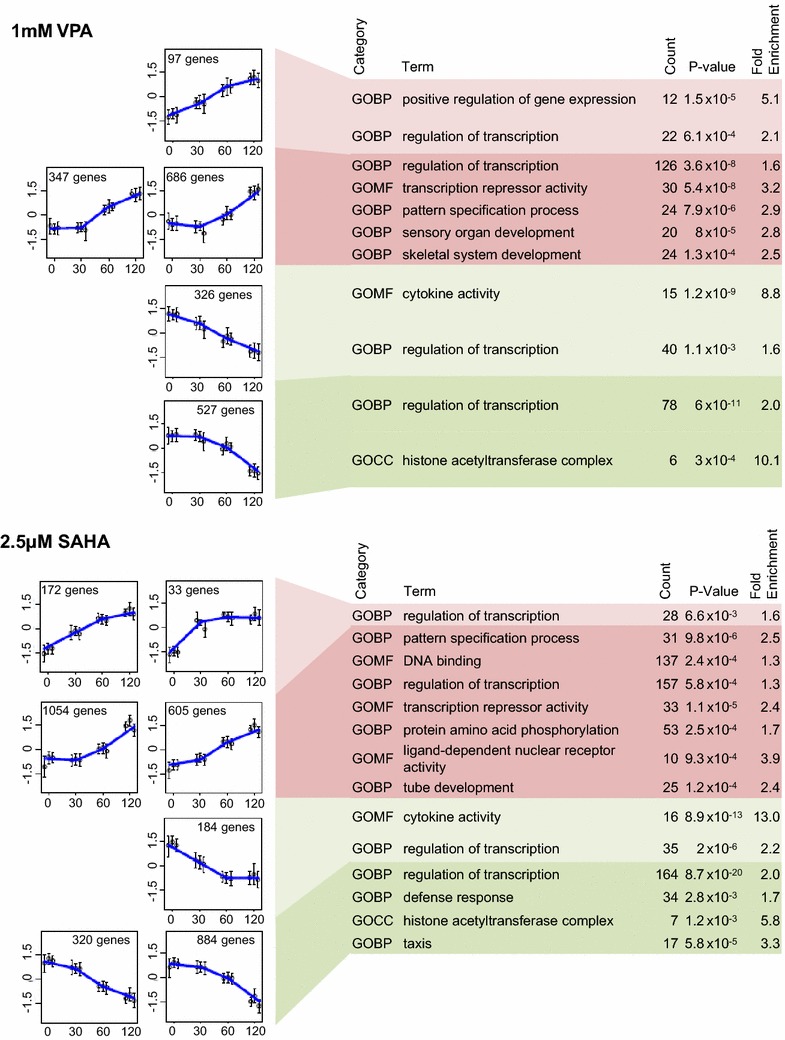
Dynamics and ontology of the transcriptional response to HDACi. Significant genes were identified by ANOVA (fold change >1.5, FDR <10 %) and clustered by SOTA. The four major clusters represent genes that were up- or down-regulated, either with or without a delay. Minor clusters, containing less than 1 % of all genes, are not shown. Each subset of genes was subject to ontological analysis by DAVID. For each significant *annotation* cluster (*enrichment score* >2) the top ontological term is shown. For each gene ontology term, *count* refers to the number of significant genes involved in that term, *fold enrichment* represents the enrichment in the significant gene list, of genes involved in that term relative to background, and the *P* value is a measure of the significance of that enrichment.

Genes were clustered into categories according to how their expression changed over time. The clustering algorithm was allowed to select the number of groups, which ranged from 4 (0.5 µM SAHA) to 10 (2.5 µM SAHA), with just 2 for 0.2 mM VPA, where only 22 genes met the selection criteria. This strategy resulted in a few groups that contained only a small number of genes and, for clarity, only those groups containing >1 % of the total number of genes analysed are shown in the figures. At every concentration except for 0.2 mM VPA, the great majority of responding genes behaved in one of four general ways, namely progressively increasing from *t*_0_, progressively decreasing from *t*_0_, increasing after an initial lag and decreasing after an initial lag. Results for 1 mM VPA and 2.5 µM SAHA are shown in Fig. 2 and for the remaining concentrations in Additional file 2. The two groups from 0.2 mM VPA treatment are shown as a heat-map in Additional file 3A.

**Fig. 3:**
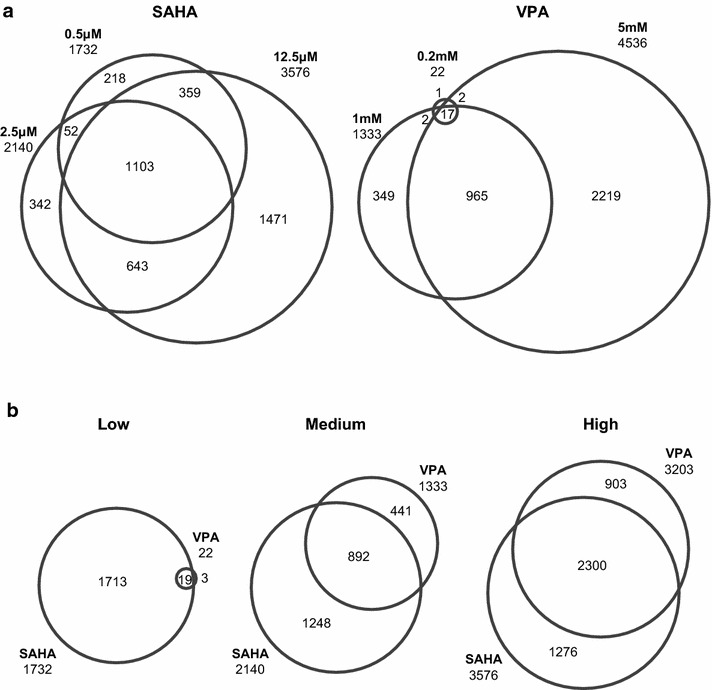
Comparison of gene populations whose transcript levels change at different concentrations of VPA and SAHA. Venn diagrams show overlap of genes responding significantly (ANOVA, fold change >1.5, FDR <10 %) to one of three concentrations of either VPA or SAHA. **a** Comparison of genes responding significantly to three different concentrations of SAHA or VPA, as indicated. **b** Comparison of significant genes responding to VPA and SAHA at each concentration (low = 0.2 mM VPA, 0.5 µM SAHA; medium = 1 mM VPA, 2.5 µM SAHA; high = 5 mM VPA, 12.5 µM SAHA).

For each inhibitor, there was a strong overlap between genes responding at the different concentrations tested (Fig. 3a) and between SAHA and VPA (Fig. 3b). For example, combining genes regulated at all doses of each inhibitor, 73 % of those genes responding to VPA also responded to SAHA. It is particularly interesting to see that of the 22 genes whose transcription is significantly affected by 0.2 mM VPA (listed in Additional file 4), 21 are also affected at higher concentrations of VPA (Fig. 3a) and 19 are affected by SAHA (Fig. 3b). This gives confidence that despite the small numbers involved and the very low dose of VPA, the changes detected are part of the same cellular response triggered at higher concentrations.

### A coordinated transcriptional response to HDACi

Ontology analysis (DAVID, [55]) was used to characterise the genes within each of the four groups identified by clustering for 1 and 5 mM VPA and all concentrations of SAHA (Fig. 2, Additional file 2). Early and late responding genes, whether up- or down-regulated and at all concentrations of both inhibitors, were highly enriched in genes involved in transcriptional regulation. These included a large number of DNA-binding zinc finger proteins (Fig. 2, Additional file 2).

There was universal down-regulation of genes encoding components of lysine acetyltransferases (KAT) complexes, always following an initial delay (Fig. 2, Additional file 2). Table 1 shows the 12 KAT complex members that were down-regulated and the extent of the observed change. Between them, the down-regulated KAT complexes acetylate all four core histones, along with an unknown number of non-histone proteins.

**Table 1:**
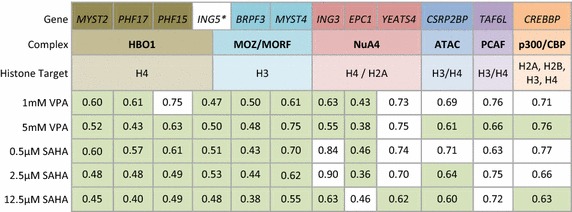
Genes encoding components of KAT complexes are consistently down-regulated by HDACi

The table shows the fold change between 0 and 120 min of genes encoding KAT complex proteins at the inhibitor concentrations shown. Statistically significant changes (ANOVA, fold change >1.5, FDR <10 %) are shaded in green. No KATs were significantly down-regulated by 0.2 mM VPA.

* *ING5* is reported to be a member of the HBO1 and MOZ/MORF complexes.

Genes within the “cytokine activity” term were rapidly down-regulated by both inhibitors (Fig. 2). Across all experiments, this category comprised 41 genes, most of which are involved in the differentiation or function of lymphocytes and other cells of the immune system (Additional file 5). They include 6 interleukins and 12 genes from the Interferon alpha (IFNA) cluster on chromosome 9. We note also that the TGF-beta superfamily gene GDF9, essential for G1-S and G2-M progression [56], and its paralogue GDF15, are consistently down-regulated (Additional file 5); this response can explain the characteristic change in cell cycle profile induced by HDACi (Additional file 1).

Developmental terms such as “pattern specification process” and “HOX genes” were up-regulated, following a lag, by both inhibitors (Fig. 2). The “pattern specification process” category included 51 genes that were significantly up-regulated in at least one of the five experiments shown in Fig. 2 and Additional file 2. Amongst these genes (listed in Additional file 6) we find 13 homeobox genes and multiple genes encoding components of the Wnt, Shh and Notch signalling pathways (6, 4 and 3 respectively).

The lowest concentration of VPA tested (0.2 mM) is within the range likely to be achieved in the body fluids of patients taking therapeutic doses of VPA [57]. By ANOVA, we identified just 22 genes responding at this dose at 30 min and thereafter. Of these, 17 were up- and 5 down-regulated (Additional files 3, 4). Amongst the down-regulated minority was EPC2, a paralogue of the HAT component EPC1 (Table 1). Of the 17 up-regulated genes, the great majority (at least 15) encode transcription factors and proteins involved in growth control and developmental signalling. In other words, the ontology closely resembles that observed at much higher concentrations.

A novel mass spectrometric approach has been used to measure the in vivo sensitivity of individual HDAC complexes (CoREST, NCoR, NURD, Sin3) to inhibitors, and has shown that they differ widely in their sensitivities to VPA, with mean Kd values from 0.6 to 13 mM [58]. In view of this, one might expect to find different gene sets responding at different VPA concentrations. Despite a careful analysis over a 25-fold concentration range, we find no evidence that the characteristic gene composition of the transcriptional response to VPA varies with inhibitor concentration.

### Early and progressive changes in histone modification in response to HDACi

We used Chromatin ImmunoPrecipitation and high-throughput sequencing (ChIP-seq) to follow changes in histone modification after 30, 60 and 120 min treatment with 1 mM VPA. We used antisera specific for H3K9ac and H4K16ac, both associated with transcriptionally active genes [27, 28], and the Polycomb-associated silencing mark H3K27me3 and its activating counterpart H3K27ac [30]. Histone modification levels were analysed using probes from 500 bp upstream to 500 bp downstream of transcription start sites (TSS).

**Fig. 4:**
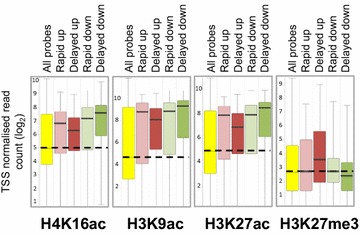
Pre-treatment histone modification levels at the TSS of genes showing specific transcriptional responses to VPA. *Box plots* show the levels of the indicated histone modifications, in untreated cells, for genes showing different types of transcriptional response to 1 mM VPA (Fig. 2), namely up- or down-regulation, with or without a delay, as indicated. Modifications were detected by ChIP-seq using chromatin from lymphoblastoid cells treated with 1 mM VPA. TSS probes were −500 to +500 bp around each TSS and were quantified by read count, normalised to the largest datastore. The *horizontal line* within each *box* represents the median value, the *upper* and *lower* extremities of the *box* represent the 75th and 25th percentile and the *whiskers* show the median ± twice the interquartile range.

**Fig. 5:**
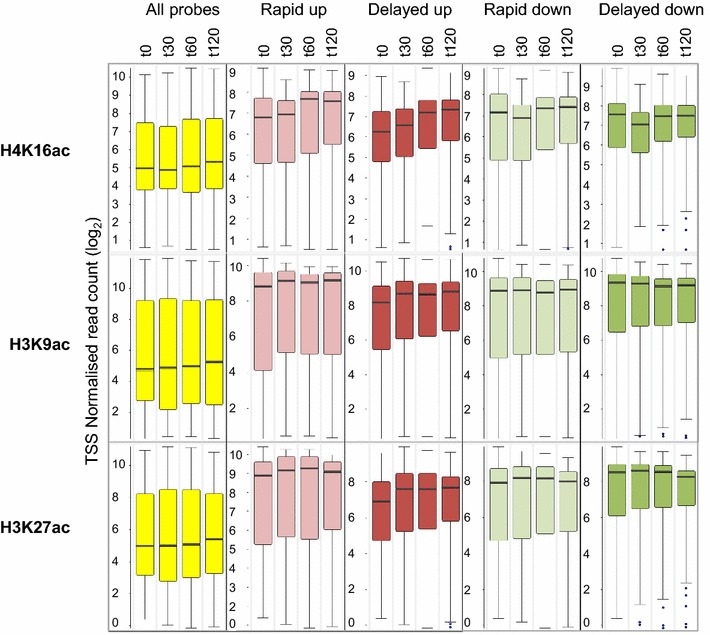
Changes over time in histone acetylation at the TSS of genes showing specific transcriptional responses to VPA. ChIP-seq was carried out on lymphoblastoid cells treated for 0, 30, 60 and 120 min with 1 mM VPA using antibodies to one of three different acetylated histones, as indicated. Changes over time are shown for each of the four categories of persistently responding genes (Fig. 2), as indicated. *Box plots*, configured as in Fig. 4, show the distribution of normalised read counts at the TSS of genes that showed significantly altered transcript levels at each time point. TSS probes were −500 to +500 bp around each TSS and were quantified by read count quantitation, normalised to the largest datastore.

We assayed acetylation at TSS of the four subsets of genes involved in the early transcriptional response, namely rapidly up or down and delayed up or down (Fig. 2). A striking initial finding was that genes involved in the progressive transcriptional response, had high basal acetylation levels. This was irrespective of the direction of transcriptional change adopted in response to HDACi (Fig. 4). In contrast, levels of H3K27me3 prior to treatment were close to the median for three of the four categories of responding genes (Fig. 4). The exception was genes that were up-regulated after a delay, which had relatively high levels of H3K27me3 in untreated cells. It is interesting that for this category of genes, levels of acetylation at all three histone lysines tested, were lower (though still well above the untreated median) than for the other three categories of responding genes (Fig. 4). Changes in acetylation following exposure to HDACi, were small. Genes that were up-regulated, whether immediately or with a delay, showed, on average, a gradual, progressive increase in H4K16ac (Fig. 5). For the same categories of up-regulated genes, H3K9ac and H3K27ac increased slightly after 30 min, but then remained unchanged for the rest of the time-course. Down-regulated genes showed no change in H3K9ac or H3K27ac across the entire time-course and just a small and transient reduction in H4K16ac at 30 min (Fig. 5). Thus, changes in histone acetylation at the TSS of persistently responding genes are small, associated almost exclusively with up-regulated genes and tend to occur soon (30 min) after addition of the inhibitor.

**Fig. 6:**
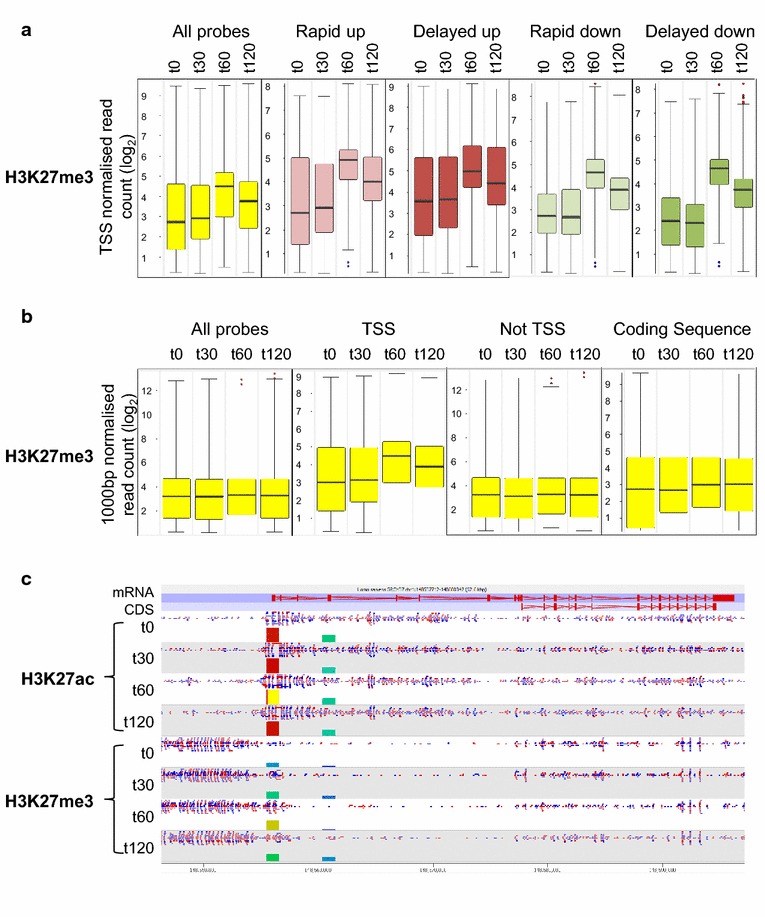
Changes over time in H3K27 tri-methylation at the TSS of genes showing specific transcriptional responses to VPA and at other genomic regions. **a** Levels of H3K27me3, after increasing time in 1 mM VPA, at the TSS of genes involved in the four categories of persistent transcriptional response (Fig. 2), as indicated. TSS probes were −500 to +500 bp around each TSS and were quantified by read count, normalised to the largest datastore. **b** Levels of H3K27me3, after increasing time in 1 mM VPA, at different genomic regions. 1,000 bp rolling windows were quantified by read count quantitation, normalised to the largest datastore. The distribution of all probes and those overlapping with TSS, not overlapping with TSS or overlapping coding sequence are shown. **c** Screenshot from SeqMonk showing changes in H3K27me3 and H3K27ac at and around the TSS of the NBPF15 gene. The positions of the gene and the coding sequence are indicated across the *top* of the panel. Forward reads are shown in *red* and reverse in *blue*. The *coloured boxes* below each data track show the position and value (box height) of each TSS probe.

Unexpectedly, the most dramatic change in histone modification in response to HDACi was in levels of H3K27me3, a modification put in place by the Polycomb silencing complex PRC2 [59]. A strong increase in H3K27me3 at TSS was first detected at 60 min and persisted at 120 min (Fig. 6a). This change was detectable across all genes (Fig. 6a, “All Probes”), and was independent of the manner in which transcription was altered by HDACi (Fig. 6a). We analysed the genomic distribution of the change in H3K27me3 with 1,000 bp rolling window probes. The increase in H3K27me3 after 60 min was seen only in windows which included TSS and not in regions classed as non-TSS or in windows overlapping other genomic features (Fig. 6b). All four sets of persistently responding genes revealed by SOTA showed increased levels of H3K27me3 at *t*_60_ and *t*_120_, but no change at *t*_30_.

The most likely explanation for HDACi-induced increase in H3K27me3 specifically at TSS, is that levels of catalytically active PRC2 have increased at TSS. This in turn could be attributed, for some genes at least, to spreading of PRC2 from adjacent, PRC2-rich, regions. Examination of genes with blocks of H3K27me3 adjacent to the TSS was consistent with this. Figure 6c shows a screenshot of the NBPF15 gene: H3K27me3 can be seen to spread over the TSS 60 min after addition of HDACi. In contrast, the distribution of H3K27ac in the gene body shows no sign of spreading (Fig. 6c), in line with the general lack of effect of HDACi on acetylation of chromatin at and around TSS (Fig. 5). We note that, in the treated cell population, the TSS contains both H3K27me3 and H3K27ac. This could be due to the existence of separate cell populations (i.e. with either methylated or acetylated TSS) or to the presence of both modifications on the same chromatin fragment. Further experimentation using this model system should allow this interesting issue to be resolved for specific TSS.

### Inhibition of the EZH1/2 methyltransferases modifies the transcriptional response to VPA

**Fig. 7:**
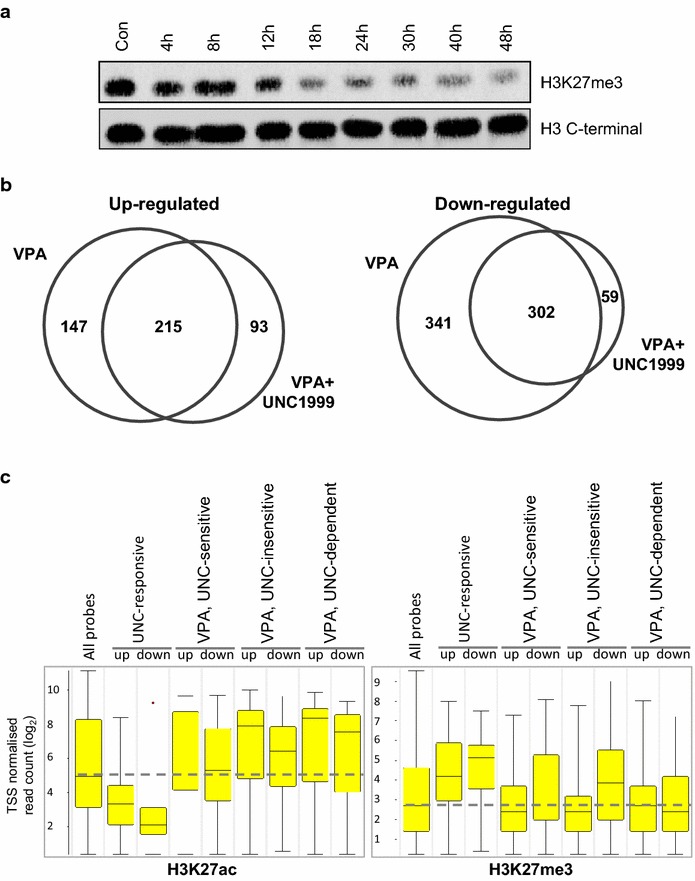
How inhibition of EZH1/2 changes the transcriptional response to VPA. Human lymphoblastoid cells were treated for 2 hours with either 1mMVPA, 3 µM UNC1999 (an inhibitor of the methyltransferase activity of EZH1/2) or both compounds together. **a** Western blot showing changes in H3K27me3 levels in LCLs treated with 3 µM UNC1999 for the indicated times. **b** Venn diagrams comparing the response to VPA alone and VPA + UNC1999, and identifying genes that are UNC1999 sensitive, UNC1999-insenstive, or whose transcription changes *only* in the presence of UNC1999 (UNC1999-dependent), as indicated. For each treatment, gene expression changes were compared to untreated cells. Significant genes were determined by *t* test (fold change >1.5, *P* < 0.05). **c** Pre-treatment levels of H3K27ac and H3K27me3 at the TSS of genes whose transcription changes (up or down as specified) in response to VPA and/or UNC1999. *Box plots* show results for all TSS (All probes, LH side), and for genes responding to UNC1999 alone (UNC-responsive), VPA only in the absence of UNC1999 (UNC-sensitive), VPA with or without UNC1999 (UNC-insensitive) and VPA only in the presence of UNC1999 (UNC-dependent). *Box plots* are configured as in Fig. 4.

To explore the possible involvement of EZH2 activity in establishing the transcriptional response to HDACi, we used UNC1999 [60, 61], an inhibitor of both EZH2 and its close homologue EZH1. Inhibition of both enzymes is important in view of evidence from mouse knockout studies that EZH1 can substitute for EZH2 to maintain PRC2 function [32, 62]. We tested transcriptional change after treating for 120 min with 1 mM VPA alone, 3 µM UNC1999 alone and VPA + UNC1999. At 3 µM, UNC1999 led to a progressive reduction in global levels of H3K27me3 (Fig. 7a) but had no effect on cell viability, either alone or in combination with 1 mM VPA (Additional file 7). It should be noted that the reduction in global levels of H3K27me3 was slow, occurring over days. Effects observed after only 120 min are likely to be due to the inhibition of de novo H3K27 tri-methylation at specific loci rather than a general reduction in H3K27me3 levels. UNC1999 on its own had a small transcriptional effect after 120 min (32 genes up, 31 down) but had a significant effect on the transcriptional response to VPA. The numbers of genes up- and down-regulated by VPA and UNC1999 are shown in Fig. 7. Of 362 genes down-regulated by VPA in this experiment, 147 (41 %) were UNC1999 sensitive (i.e. their down-regulation was prevented by UNC1999), while 215 (59 %) were UNC1999 insensitive. These two populations had different ontologies (Additional file 8). Cytokine and apoptosis-related genes were UNC1999 sensitive (EZH2 dependent), while genes involved in transcriptional regulation and, importantly, KAT complex genes, were UNC1999 insensitive (EZH2 independent). Ninety-three genes were down-regulated only by VPA plus UNC, indicating that their ability to respond to VPA is normally suppressed by PRC2-mediated H3K27 methylation. Of 643 genes up-regulated by VPA in this experiment, 341 (53 %) were UNC1999 sensitive (EZH2 dependent) and 302 (47 %) were UNC1999 insensitive (EZH2 independent). As with down-regulated genes, the ontologies of the two groups differed, with transcription related terms again confined to the UNC1999 insensitive group. Developmental terms were found in both groups, but were much more prominent amongst UNC1999 sensitive (EZH2 dependent) genes (Fig. 7b). A relatively small number of genes, 59, were up-regulated only by VPA plus UNC, indicating that their up-regulation by VPA is normally suppressed by PRC2.

These results show that ongoing EZH1/2 activity is essential for the VPA-induced change in expression of some categories of gene. Surprisingly, the results show that EZH1/2 can be required for either activation or silencing, depending on the genes involved. We used ChIP data to ask whether the effects of EZH1/2 inhibition were influenced by the H3K27 modification status prior to treatment, but found little evidence for this. Genes whose transcription was changed (up or down) by UNC1999 *alone* had relatively low levels of H3K27ac and high levels of H3K27me3, indicating silenced or lowly expressed genes (Fig. 7c). Apart from this, all VPA-responsive genes, irrespective of their EZH1/2 dependence, had levels of H3K27ac above the population median and levels of H3K27me3 close to the median (Fig. 7c).

## Discussion

### Adaptive strategies

We have shown that the transcriptional response that develops during the first 2 h of exposure to HDACi involves a restricted number of genes, largely within specific functional categories. These categories indicate a coordinated transcriptional response that allows the cell, first, to survive, and then to adapt to life in the presence of HDACi. A prominent feature of the early HDACi response is the universal down-regulation of genes encoding KAT complex components. This will mitigate the hyperacetylation induced by HDACi, and explains both the return to normal levels of acetylation after prolonged culture in HDACi (e.g. [63]) and the very early finding that histone acetylation levels fell rapidly to below pre-treatment levels when HTC cells were released from prolonged butyrate inhibition [17].

Other gene sets with characteristic patterns of transcriptional change provide additional clues to the nature of the adaptive response. The rapidly and consistently down-regulated cytokine category was particularly enriched in genes involved in growth control, such as GDF9 and its paralogue GDF15. GDF9 is necessary for cell cycle progression, mediating both the G1-S transition and passage through G2-M [56]. GDF9/GDF15 down-regulation may explain the slowed growth and G1 and G2M blocks caused by HDACi in LCLs (Additional file 1A) and other cell types [64]. Slowed growth will protect the cell against long-term genome damage caused by replicative stress through S-phase and chromosome mis-segregation at mitosis.

In order to function and grow (albeit slowly) in the presence of HDACi, the cell must establish a new pattern of gene expression supported by a modified epigenome. Our results show *down*-*regulation* of genes potentially involved in determining the lymphoid phenotype of the LCL, such as interleukins and alpha interferons and *up*-*regulation* of genes required for a change of phenotype, including homeobox genes and components of the Wnt and Notch signalling pathways. This change need not involve complete loss of the original lymphoid phenotype, or differentiation along a pre-defined lineage, and we see no evidence for this. Indeed, there are likely to be many different epigenomes and transcriptomes that are compatible with survival and growth in the presence of HDACi. The changes we observe increase the chances of one of these states being reached by any single cell. Longer term, cells whose modified transcriptomes allow them to cope most effectively with the prevailing environment, will come to dominate the cell population through normal selection processes.

### The evolutionary necessity of an adaptive response to HDACi

Many HDACi are natural products, usually produced by bacteria, and are widely present in the environments encountered by eukaryotic cells [65, 66]. Short-chain fatty acids are common products of bacterial metabolism and are present at mM concentrations in the large intestines of humans and other mammals [67, 68]. HDACi such as Trichostatin A, Trapoxin and Depsipeptide, are bacterial antimicrobials that kill eukaryotic micro-organisms (such as Aspergillus) competing for the same resources [69]. Killing of competitors and predation are common in microbial communities [70]. Over vast periods of evolutionary time since their first emergence, eukaryotes must have evolved strategies to protect themselves, and their uniquely eukaryotic chromatin-based epigenetic systems, from HDACi secreted by competing prokaryotes. This evolutionary background, and the fact that humans and other higher eukaryotes continue to be exposed to environmental HDACi [68], provides a convincing rationale for the existence of the protective response uncovered by our experiments.

### Histone modifications as determining factors in the transcriptional response to HDACi

Our results show that the way in which genes initially respond to HDACi is closely associated with their histone modification status prior to treatment. Genes that are rapidly up- or down-regulated, some of which form part of the proposed adaptive response, are marked by high levels of H3 and H4 acetylation, even though their transcript levels are close to the population mean. Following HDACi treatment, despite the rapid increase in histone acetylation detected by Western blotting, changes in acetylation at and around TSS were modest at best, and often absent. This can be attributed, in part to the initially high levels of histone acetylation of HDACi-responsive genes, but still indicates that increased histone acetylation is not an immediate driver of the transcriptional changes we detect. It seems that high levels of histone acetylation provide a chromatin context that allows genes to change their transcription level (up- or down-) in response to the appropriate signals.

Unexpectedly, the most striking change in histone modification over the first 120 min of treatment with HDACi, was an increase in H3K27me3 at TSS. We detected no increase in transcripts encoding PRC2 core components, and it seems likely that our findings reflect the redistribution of existing PRC2 complexes to TSS [71]. The processes by which PRC2 is directed to its target sites, and by which it is retained there, are complex and still incompletely understood [72, 73]. H3K27me3 is necessary for stable PRC2 binding and maintenance of silencing [74]. H3K27 methylation can be blocked by pre-existing acetylation of the same residue, and this modification is antagonistic to silencing [30, 32]. In view of this, it is interesting that we were able to show that blocks of H3K27ac extending from the gene body into the TSS did not prevent the spreading of H3K27me3 into the TSS from enriched upstream regions. Within treated populations, the nucleosome at the TSS contained both acetylated and/or tri-methylated H3K27. While it is theoretically possible that the two different H3 modifications are carried on a single nucleosome [75], it is more likely that that result reflects a mixed population of cells, with some having H3K27me3 on the single TSS nucleosome and others H3K27ac. It is clear that VPA does not induce any detectable spreading or enhancement of blocks of H3K27ac, in line with its modest effects on gene-proximal histone acetylation in general.

### The role of Polycomb mediated gene regulation

Phosphorylation of H3 at serine 28, catalysed by MSK1/2, can displace PRC2, presumably by disrupting binding to the adjacent, methylated residue [76, 77]. H3S28ph levels have been shown to increase at the promoter regions of a specific subset of genes that are transcriptionally activated when quiescent 3T3 cells are stimulated back into growth [78]. This allows Polycomb-silenced genes to be reactivated without the need to demethylate H3K27. This finding is relevant to our results in that the antibody to H3K27me3 used for these experiments, while highly specific for methylated H3K27 in peptide binding assays, binds only weakly to peptides that are also phosphorylated at K28 [79]. Thus, it is possible that the increased, TSS-specific antibody binding that occurs after 60 min exposure to HDACi, reflects unmasking of pre-existing H3K27me3 by de-phosphorylation of H3K27me3S28ph. We cannot discount this possibility, but regard it as unlikely, not least because the precisely timed increase in H3K27me3 that we observe occurs at TSS, irrespective of their transcriptional status, contrary to the selective phosphorylation of H3S28 at active TSS [78]. However, the functional outcome will be the same in each case, namely enhanced gene silencing through H3K27me3-mediated binding of PRC2.

Surprisingly, genes that were either up- or down-regulated over the first 120 min were distinguished from one another not by H3/H4 acetylation, which was high for both, but by H3K27me3, which was elevated, in untreated cells, only for genes up-regulated following a delay. Genes that carry a combination of histone modifications normally associated with active or silent chromatin states were first identified in embryonic cells [80, 81]. These “bivalent” genes were shown to correspond largely to genes that were poised to respond to developmental signals [80–83]. It may be that HDACi selectively activate such poised, bivalent genes. This is consistent with our ontology analysis which shows enrichment of up-regulated genes in terms relating to transcriptional regulation and development. We also note that the regulatory effects of PRC2 can be modulated by histone modifications associated with active chromatin, such as H3K4me3 [31], raising the possibility that PRC2 effects might differ depending on levels of histone acetylation.

## Conclusions

Cells respond to HDACi with a transcription based, adaptive response that allows them to survive in the presence of the inhibitor. The response includes the universal down-regulation of KAT complex components, a change that will diminish and eventually eliminate protein hyperacetylation caused by HDAC inhibition, and the up-regulation of developmental regulators that adjust transcription to accommodate inhibitor-induced epigenomic changes. Genes whose expression is sensitive to HDACi are consistently pre-marked by high levels of H3 and H4 acetylation. We find little evidence that transcriptional changes are driven by further increases in histone acetylation at gene control regions, but there is a dramatic, across-the-board increase in H3K27me3 at transcription start sites. Inhibition of the methyltransferase EZH1/2 alters the transcriptional response, confirming the functional involvement of the Polycomb complex PRC2. Collectively, our results support the conclusion that the transcriptional response to HDACi, including activation or mobilisation of PRC2, is driven by increased acetylation of specific non-histone proteins, with histone acetylation providing a chromatin context that allows transcriptional change. Given the potential importance of the survival response in determining the resistance or sensitivity of cancer cells to therapeutic doses of HDACi, the identification of these proteins is a key objective.

## Methods

### Cell culture

The work described here used two long-established human lymphoblastoid cell lines (LCL), GM12878 (Coriell Institute, see [28]) and AH-LCL (produced in-house in the course of previous work by Rowe and colleagues [84]). Lines were maintained at 37 °C, 5 % CO_2_ in RPMI 1640 medium, 10 % foetal bovine serum, supplemented with l-glutamine (2 mM) and penicillin/streptomycin (all reagents from Life Technologies). A stock solution of sodium valproate (VPA, Sigma) was prepared at 1 M in water and stocks of suberoylanilide hydroxamic acid (SAHA, Sigma) were prepared at 0.5, 2.5 and 12.5 in DMSO. Cells were treated at 0.2, 1 and 5 mM VPA and 0.5, 2.5 and 12.5 µM SAHA such that DMSO concentration in SAHA-treated cells was always 0.1 %. A stock solution of UNC1999 was prepared at 3 mM in DMSO and cells were treated with 3 µM UNC1999.

### Antibodies

Rabbit polyclonal antisera to H4K16ac (R251), H3K9ac (R607) and H3K4me3 (R612) were raised in-house by immunisation with synthetic peptides conjugated to ovalbumin as previously described [85]. The antibody to H3K27me3 was from Millipore (07-449) and to H3K27ac from Abcam (ab4729). Antibody specificities was assayed by inhibition ELISA and checked by Western blotting.

### Western blotting

Histones were extracted from ES cells by acid extraction and analysed by electrophoresis in 15 % SDS–polyacrylamide gels and western blotting as previously described [4]. Protein loading was confirmed by Ponceau S staining before proteins were probed with appropriate primary antibodies. Primary antibody binding was detected by fluorescent-tagged anti-rabbit IgG secondary antibody (Rockland) and detected by scanning (Odyssey system; LI-COR, Cambridge, UK).

### FACS cell cycle analysis

Treated and control cells were washed in PBS and fixed in ice cold 90 % ethanol at −20 °C for at least 30 min. Fixed cells were washed in PBS and resuspended in propidium iodide staining buffer [PBS with 20 µg/ml propidium iodide (Sigma), 100 µg/ml RNase A (Life Technologies), 0.1 % NP40, 5 µg/ml tri-sodium citrate (Sigma), 5 % foetal bovine serum (Life Technologies) and 0.02 % sodium azide (Sigma)]. Cells were analysed on a Cyan ADP Flow Cytometer using Summit software v4.3 (Beckman Coulter), gating for forward and side scatter and pulse width to isolate single cells. Apoptotic populations in untreated or treated LCLs were determined using the Annexin V Apoptosis Detection Kit—APC (eBioscience) according to the manufacturer’s instructions.

### Microarray expression analysis

All microarray experiments were carried out in biological triplicates. All microarray reagents are from Roche Nimblegen unless otherwise stated. Cells were harvested by centrifugation and RNA was extracted and purified using the RNeasy kit with DNase digestion (Qiagen) according to the manufacturer’s instructions. Double stranded cDNA was synthesised using the cDNA Synthesis System, including RNase I and Proteinase K treatment followed by DNA clean up using the PCR purification kit (Qiagen). Samples were labelled with cy3 using a Nimblegen One-Colour Labeling Kit, mixed with alignment oligos and sample tracking control oligos (Nimblegen Hybridisation and Sample Tracking Control Kits) and hybridised to a 12 × 135k HD2 expression array (Roche Nimblegen, containing 3 probes per sequence for 44,049 human sequences) and scanned on a Nimblegen MS200 Microarray scanner. Data were extracted using DEVA (Roche Nimblegen) and normalised by robust multichip average in R. Direct comparisons of *t*_0_ and individual time points was carried out using *t* Tests. Genes with a *P* value smaller than 0.05 and FC larger than 1.5 have been selected for further analysis including functional annotation and network construction. The ongoing transcriptional response was identified using ANOVA in TMEV [86]. The *P* values derived from ANOVA analysis were corrected for multiple testing by performing the Benjamini–Hochberg correction in R. Differentially expressed genes were selected using a threshold of false discovery rate (FDR) smaller than 10 % with fold change greater than 1.5. In order to group genes with similar expression patterns into clusters, a SOTA analysis was performed using TMEV [86]. Each cluster that was characterised by highly co-regulated genes was then functionally annotated for GO terms using the web based tool DAVID [55]. Co-regulation networks were constructed using ARACNE and visualised in Cytoscape with a forced directed layout. The microarray data from this publication have been submitted to the GEO database (http://www.ncbi.nlm.nih.gov/geo/) and assigned the identifier GSE65297.

### Chromatin immunoprecipitation: sequencing

Immunoprecipitation of native chromatin was performed based on the method described previously [87]. Briefly, cells were lysed to release nuclei prior to micrococcal nuclease digestion. The amount of micrococcal nuclease added and digestion time were adjusted to obtain a mix of mono- and short oligo-nucleosomes, optimal for immunoprecipitation. Chromatin was pre-cleared by incubation with protein A Sepharose beads and incubated overnight with antibodies to H4K16ac, H3K9ac, H3K27ac and H3K27me3. Antibody-bound material was isolated on Protein A-Sepharose beads (Invitrogen, UK) and DNA from antibody-bound and input chromatin was purified by PCR purification kit (Qiagen). Sequencing libraries were prepared from 100 ng DNA per sample using the KAPA Library preparation kit for Illumina (Anachem). Samples were barcoded using the system described by Bronner et al. [88]. Sequencing was carried out at the West Midlands Regional Genetics Laboratory. Pooled libraries were clustered using a ‘cBot’ cluster generation system at a final concentration of 13 pM, followed by sequencing on a HiSeq 2500, in ‘rapid run’, paired-end mode (2 × 51 bp). FASTQ files were simultaneously generated and de-multiplexed using the Illumina ‘HiSeq Analysis software’ v0.9 followed by alignment using Bowtie2 [89]. Bowtie2 was run using the default parameters for ‘very-sensitive-local’ alignment mode. Crucially, this means the ‘best alignment’ is reported for every read that can be mapped; if there are two alignments that are tied, then one is randomly chosen. The output from Bowtie2 was converted to bam files and sorted using Samtools v0.1.19. ChIP-seq data was analysed using SeqMonk (Babraham Institute, http://www.bioinformatics.babraham.ac.uk/projects/seqmonk/).

## Additional files

Additional file 1: The global changes in histone modification and cell cycle profile in cells treated with HDACi. Additional file 2: SOTA and ontological analysis of the transcriptional response to 5 mM VPA, 0.5 µM SAHA and 12.5 µM SAHA. Additional file 3: The characteristics of genes responding to 0.2 mM VPA. Additional file 4: All genes responding significantly to 0.2 mM VPA. Additional file 5: Genes that are down-regulated by HDACi and are involved in “cytokine activity”. Additional file 6: A table showing “pattern specification process genes” that are up-regulated by HDACi treatment. Additional file 7: The effect on the cell cycle and apoptosis of VPA and UNC 1999 treatment. Additional file 8: Ontology analysis of VPA-responsive genes based on their sensitivity to UNC1999.
