# Supplementary material for: Cells adapt to the epigenomic disruption caused by histone deacetylase inhibitors through a coordinated, chromatin-mediated transcriptional response
Source: Epigenetics Chromatin. 2015 Sep 16;8:29. doi: 10.1186/s13072-015-0021-9 (PMC4572612; doi:10.1186/s13072-015-0021-9)

Additional Data File 1 - The effect of HDACi on global histone acetylation levels and cell cycle progression.

A Human lymphoblastoid cells (AH LCL) were exposed to VPA or SAHA for the times and at the concentrations indicated and histone acetylation levels assessed by western blotting. Graphs represent quantification of the images by densitometry.

B AH LCL cells were treated with HDACi for 24 hours, fixed in ethanol and stained with propidium iodide. The percentage of cells in each cell cycle phase is shown. The bar charts show the mean ± standard error from three independent experiments. *Represents P<0.05.

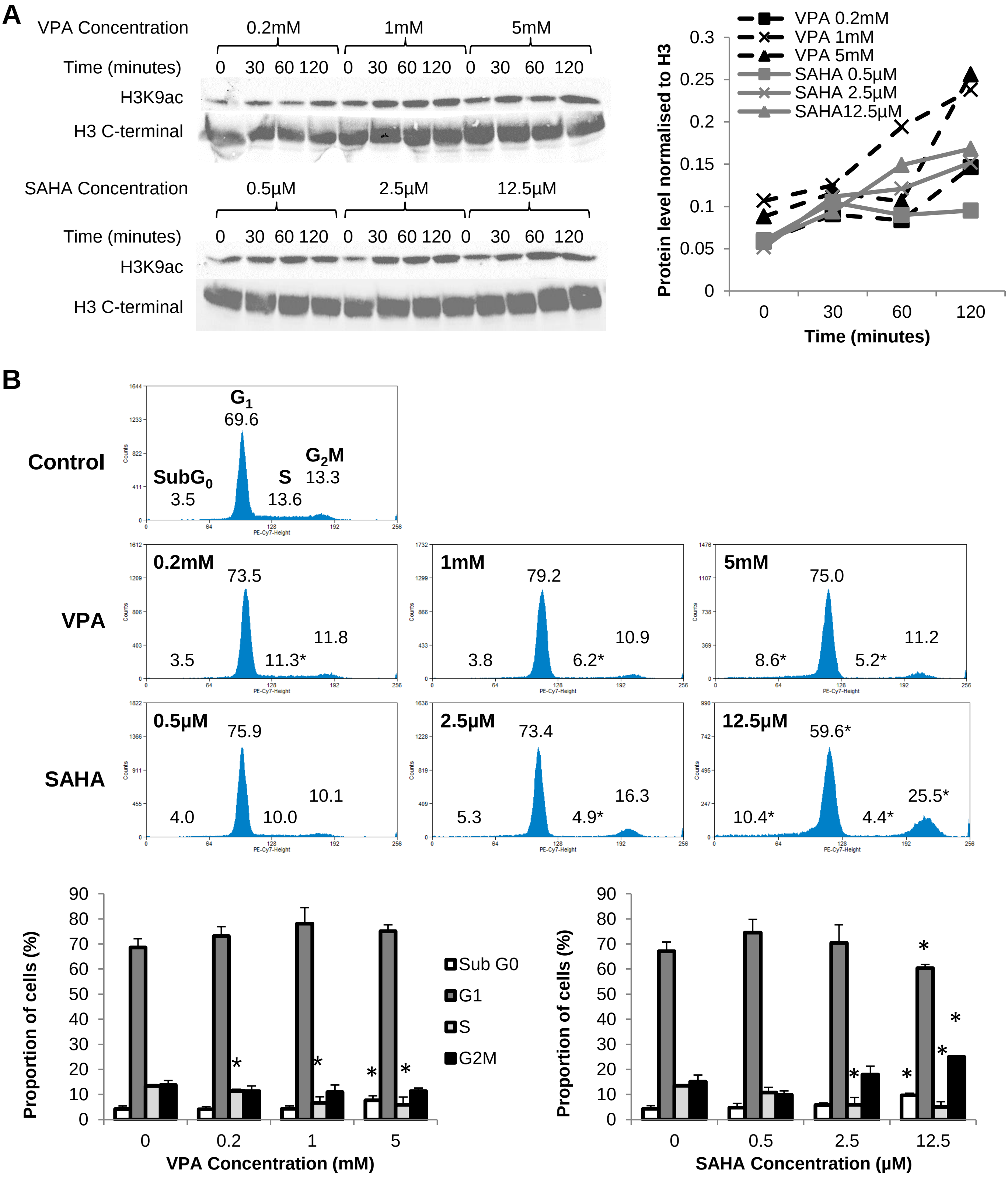

Supplement: Additional file 1: — The global changes in histone modification and cell cycle profile in cells treated with HDACi. [file 13072_2015_21_MOESM1_ESM.pdf]
