# Supplementary material for: Cells adapt to the epigenomic disruption caused by histone deacetylase inhibitors through a coordinated, chromatin-mediated transcriptional response
Source: Epigenetics Chromatin. 2015 Sep 16;8:29. doi: 10.1186/s13072-015-0021-9 (PMC4572612; doi:10.1186/s13072-015-0021-9)
Supplement: Additional file 2: — SOTA and ontological analysis of the transcriptional response to 5 mM VPA, 0.5 µM SAHA and 12.5 µM SAHA. [file 13072_2015_21_MOESM2_ESM.pdf]

Additional Data File 2 - the transcriptional response to HDACi

Significant genes were identified by ANOVA (fold change >1.5, FDR<10%) and clustered by SOTA. Clusters were combined as shown to represent rapid or delayed up- and down-regulation. For each cluster the top significant ontological terms (enrichment score >2) are shown.

A Responses to 1mM VPA

B Responses to 0.5 and 12.5µM SAHA

A

5mM VPA

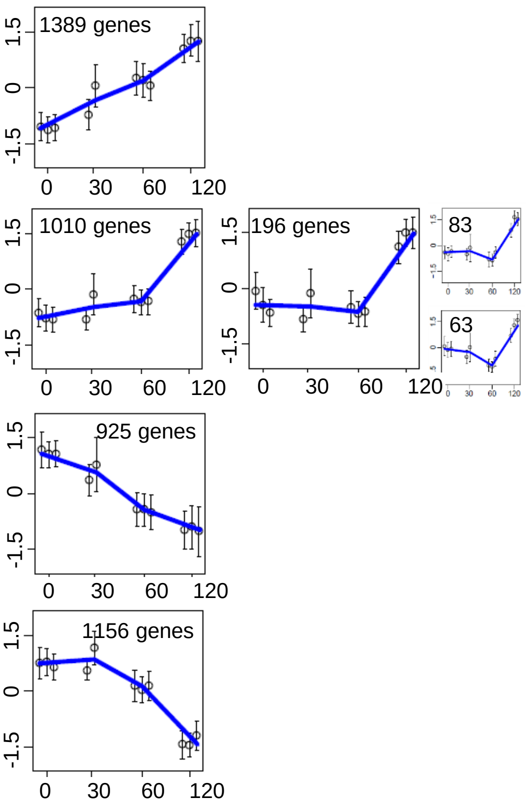

Category	Term	Count	P-value	Fold Enrichment
GOBP	regulation of transcription	138	4.1×10^{-4}	1.3
GOBP	positive regulation of transcription	38	2.7×10^{-3}	1.7
GOBP	pattern specification process	28	7.8×10^{-6}	2.6
GOBP	multicellular organism reproduction	35	9.8×10^{-4}	1.8
GOBP	endocytosis	21	4.8×10^{-4}	2.4
GOMF	ligand-dependent nuclear receptor activity	8	6.6×10^{-3}	3.6
SMRT	ZnF C2H2	76	3.5×10^{-21}	3.3
GOBP	regulation of transcription	119	2.8×10^{-13}	1.9
GOMF	cytokine activity	31	5.8×10^{-16}	6.5
GOBP	defense response	36	2.1×10^{-6}	2.4
GOMF	growth factor activity	14	1.6×10^{-4}	3.6
GOBP	regulation of transcription	126	1.8×10^{-7}	1.5
GOBP	defense response	30	1.9×10^{-2}	1.5
GOCC	histone acetyltransferase complex	10	6.6×10^{-6}	7.4
GOBP	taxis	20	6.5×10^{-7}	4.0
GOMF	glucuronosyltransferase activity	6	5.2×10^{-4}	8.6

B

0.5μM SAHA

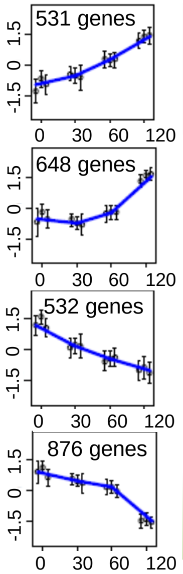

Category	Term	Count	P-Value	FE
GOBP	regulation of transcription	65	1×10^{-3}	1.5
GOMF	phospholipid binding	10	2.3×10^{-3}	3.5
GOCC	Golgi apparatus	23	1.9×10^{-3}	2.0
GOMF	glucose transmembrane transporter activity	3	2.4×10^{-2}	12.4
GOBP	pattern specification process	22	1.6×10^{-7}	4.0
GOMF	steroid hormone receptor activity	7	3.8×10^{-4}	7.2
GOMF	DNA binding	74	2.2×10^{-5}	1.6
GOBP	regulation of transcription	77	6.3×10^{-4}	1.4
GOBP	sensory organ development	15	2.9×10^{-4}	3.2
GOBP	cell fate commitment	12	1.4×10^{-4}	4.2
GOMF	cytokine activity	27	9.2×10^{-17}	8.4
GOBP	regulation of transcription	72	6.2×10^{-8}	1.8
GOBP	transcription	94	2.4×10^{-10}	1.9
GOBP	defense response	28	1.3×10^{-3}	1.9
GOMF	transcription regulator activity	51	1.9×10^{-3}	1.5
GOCC	histone acetyltransferase complex	7	4.2×10^{-4}	7.1
GOBP	chromatin modification	16	2.1×10^{-3}	2.5

12.5μM SAHA

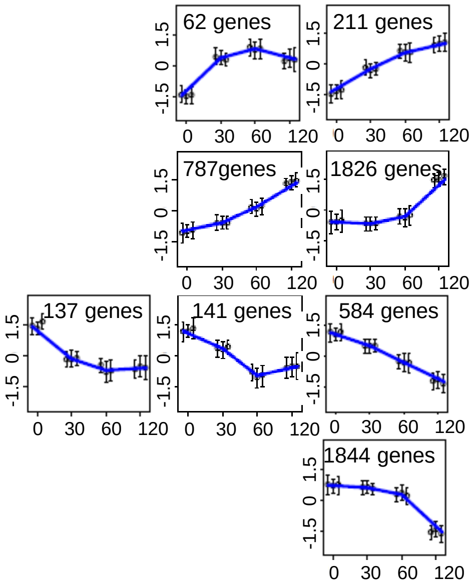

Category	Term	Count	P-Value	FE
GOMF	transcription repressor activity	9	3.8×10^{-3}	3.5
GOBP	regulation of transcription	36	7.5×10^{-4}	1.7
GOBP	pattern specification process	37	4.7×10^{-5}	2.1
GOCC	anchored to membrane	32	1.5×10^{-5}	2.3
GOBP	cell motility	38	3.8×10^{-4}	1.8
GOBP	ossification	19	6.3×10^{-4}	2.5
GOCC	intrinsic to plasma membrane	110	3.8×10^{-5}	1.5
GOBP	Wnt receptor signaling pathway	21	5.5×10^{-4}	2.3
GOBP	endocytosis	30	3.7×10^{-4}	2.0
GOMF	transcription repressor activity	36	1.2×10^{-3}	1.8
GOBP	regulation of transcription	133	2.9×10^{-22}	2.2
GOMF	cytokine activity	30	2.6×10^{-15}	6.4
GOBP	regulation of transcription	201	9×10^{-14}	1.6
GOMF	glucuronosyltransferase activity	8	4.4×10^{-5}	7.7
GOCC	integral to plasma membrane	74	1.3×10^{-4}	1.6
GOBP	defense response	45	4.3×10^{-3}	1.5
GOMF	C-C chemokine receptor activity	7	5.6×10^{-5}	9.3
GOCC	histone acetyltransferase complex	10	1.4×10^{-4}	5.0
GOBP	chromatin modification	23	1.1×10^{-2}	1.8
