# Supplementary material for: Cells adapt to the epigenomic disruption caused by histone deacetylase inhibitors through a coordinated, chromatin-mediated transcriptional response
Source: Epigenetics Chromatin. 2015 Sep 16;8:29. doi: 10.1186/s13072-015-0021-9 (PMC4572612; doi:10.1186/s13072-015-0021-9)

Additional Data File 3 - Characteristics of genes responding to 0.2mM VPA.
A Heatmap showing SOTA clustering of significant gene expression profiles (ANOVA, Fold-change>1.5, FDR<10%).
B Initial histone modification level distributions for each histone modification at the TSS of genes responding to 0.2mM VPA compared to all TSS. TSS probes were -500 to +500bp around each TSS and were quantified by read count quantitation, normalised to the largest datastore.

A

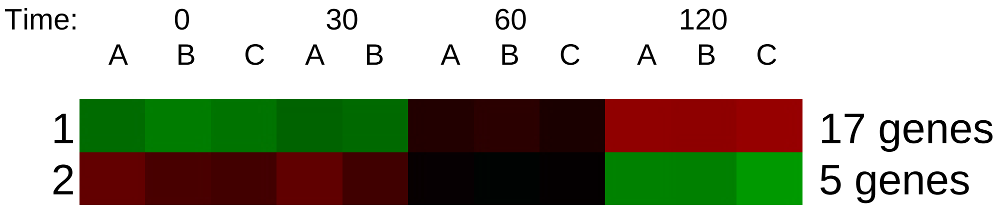

B

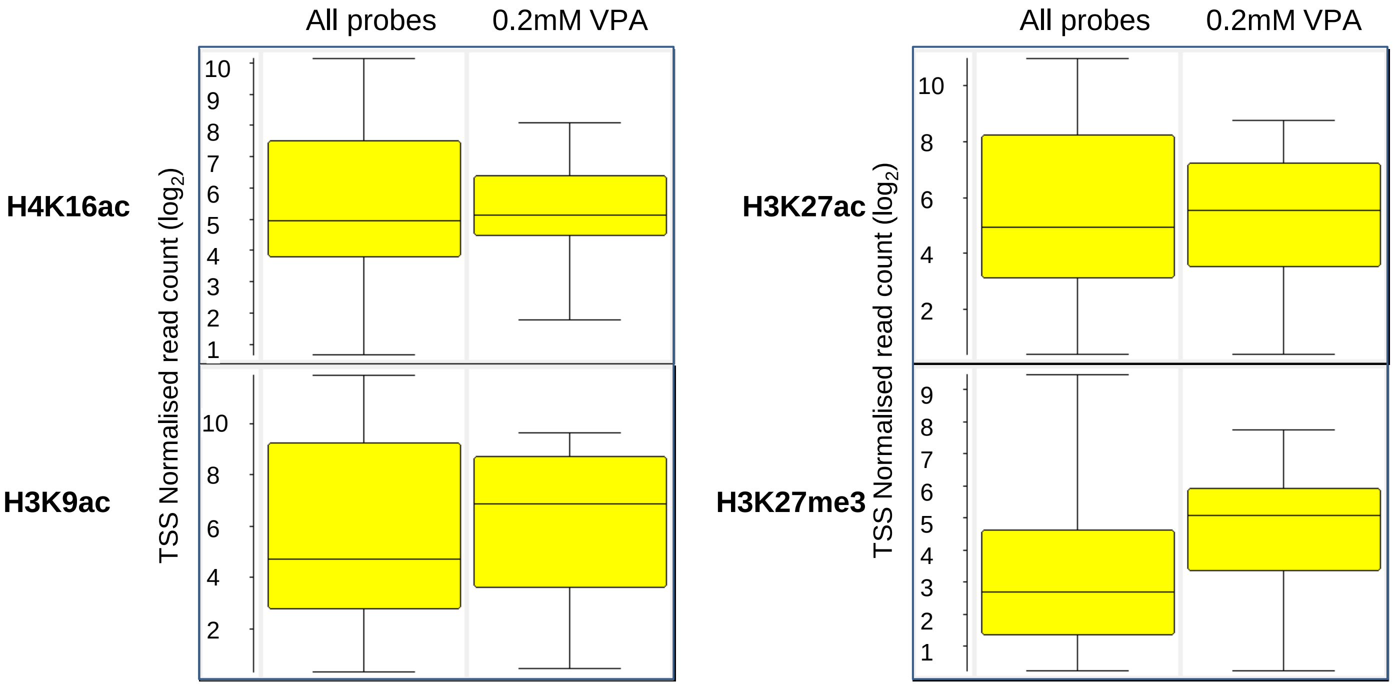

Supplement: Additional file 3: — The characteristics of genes responding to 0.2 mM VPA. [file 13072_2015_21_MOESM3_ESM.pdf]
