# Supplementary material for: Cells adapt to the epigenomic disruption caused by histone deacetylase inhibitors through a coordinated, chromatin-mediated transcriptional response
Source: Epigenetics Chromatin. 2015 Sep 16;8:29. doi: 10.1186/s13072-015-0021-9 (PMC4572612; doi:10.1186/s13072-015-0021-9)
Supplement: Additional file 4: — All genes responding significantly to 0.2 mM VPA. [file 13072_2015_21_MOESM4_ESM.docx]

**Additional Data File 4** - Genes whose expression is significantly changed after 30 min in 0.2M VPA

Significant genes were determined by ANOVA (P<0.05, FDR<10%) TF: transcription factor.

| Gene Name | Function and Notes | Refs |
| --- | --- | --- |
| ANKRD34 | Ankyrin Repeat Domain |  |
| ATXN7L2 | Unknown-Ataxin-Like, altered exp’n in NSC lung cancer | 1 |
| BTN1A1 | Milk lipid secetion (butyrophilin), binds fatty acids | 2 |
| DLX1 | TF, brain development | 3 |
| EPC2 | Enhancer of Polycomb. HAT complex subunit (NuA4, Tip60) | 4 |
| FGD4 | Actin binding, cell shape, MAPK8 regul’n. Mutated in Charcot-Marie-Tooth disease | 14 |
| FGF9 | Fibroblast Growth Factor family. Male sexual development? |  |
| GAS1 | Cell cycle progression, Hedgehog signalling | 12 |
| IPF1 | TF, dev’tal regulator, pancreas. Butyrate Induced in mESC | 5 |
| KCNJ2 | Membrane protein, K channel. Confers neuron excitability |  |
| KIAA1161 | Unknown, protein coding |  |
| KLF9 | TF, growth control, regulates p53 | 6 |
| LBX1 | TF, neural development | 7 |
| LOC113230 | Unknown, protein coding |  |
| NRIP1 | Nuclear receptor | 8 |
| OTX1 | TF, homeodomain protein, Bicoid sub-group |  |
| PLAG1 | TF, activates many genes, Zn finger, implicated in Pleiomorphic Adenoma | 13 |
| RAB3A | Signalling, Low MW G-protein | 9 |
| SH2D4B | T-cell signalling. SH2 domain Adaptor protein | 10 |
| TRIM36 | Ubiquitin ligase, Tripartite Motif. Cell cycle prog, development |  |
| USP27X | Ubiquitin-specific peptidase |  |
| ZNF333 | DNA binding. Double KRAB domain | 11 |

References

1. Wu X et al Cancer Res 2013
2. LaRocca J et al. PLoS One 2011
3. Dai X et al Eur J Neurosci 2014
4. Huang X et al Leukemia 2014
5. Goicoa S et al Cloning Stem Cells 2006
6. Sun J et al Cancer Lett 2014
7. Huang M et al Dev Biol 2008
8. Greiner EF et al PNAS 2000
9. Lodhi SS et al Mol Biol Rep 2014
10. Lapinski PE et al Immunol Rev 2009
11. Jing Z et al J Biochem Mol Bio 2004
12. Mathew E Cell Rep 2014
13. Tang Q et al PLoS One 2013
14. Horn M Brain 2012
