# Supplementary material for: Cells adapt to the epigenomic disruption caused by histone deacetylase inhibitors through a coordinated, chromatin-mediated transcriptional response
Source: Epigenetics Chromatin. 2015 Sep 16;8:29. doi: 10.1186/s13072-015-0021-9 (PMC4572612; doi:10.1186/s13072-015-0021-9)
Supplement: Additional file 5: — Genes that are down-regulated by HDACi and are involved in “cytokine activity”. [file 13072_2015_21_MOESM5_ESM.docx]

**Additional Data File 5 - Down-regulation of cytokine activity in response to HDACi treatment.**

The table shows genes encoding proteins involved in cytokine activity that are down-regulated at the inhibitor concentrations shown.

| Gene | 1mM VPA | 5mM VPA | 0.5 µM SAHA | 2.5 µM SAHA | 12.5 µM SAHA |
| --- | --- | --- | --- | --- | --- |
| AREG |  |  | 🡫 |  |  |
| BMP4 | 🡫 | 🡫 | 🡫 | 🡫 | 🡫 |
| CCL3 | 🡫 | 🡫 |  | 🡫 | 🡫 |
| CCL3L1 | 🡫 | 🡫 |  |  | 🡫 |
| CCL3L3 | 🡫 | 🡫 |  |  |  |
| CCL4 | 🡫 | 🡫 | 🡫 |  | 🡫 |
| CCL4L1 | 🡫 |  |  |  |  |
| CCL4L2 | 🡫 | 🡫 | 🡫 | 🡫 | 🡫 |
| CLCF1 |  | 🡫 | 🡫 |  | 🡫 |
| CNTF |  |  |  |  | 🡫 |
| CSF1 |  | 🡫 | 🡫 |  |  |
| CSF3 |  |  | 🡫 |  |  |
| CX3CL1 | 🡫 | 🡫 |  |  |  |
| GDF9 |  | 🡫 | 🡫 |  | 🡫 |
| GDF15 | 🡫 | 🡫 | 🡫 | 🡫 | 🡫 |
| IFNA1 |  | 🡫 | 🡫 | 🡫 | 🡫 |
| IFNA2 | 🡫 | 🡫 | 🡫 | 🡫 | 🡫 |
| IFNA4 |  | 🡫 | 🡫 | 🡫 | 🡫 |
| IFNA5 |  | 🡫 | 🡫 |  |  |
| IFNA6 | 🡫 | 🡫 | 🡫 | 🡫 | 🡫 |
| IFNA7 |  | 🡫 | 🡫 |  | 🡫 |
| IFNA8 |  | 🡫 | 🡫 | 🡫 | 🡫 |
| IFNA10 |  | 🡫 | 🡫 | 🡫 | 🡫 |
| IFNA13 | 🡫 | 🡫 | 🡫 | 🡫 | 🡫 |
| IFNA14 | 🡫 | 🡫 | 🡫 | 🡫 | 🡫 |
| IFNA17 | 🡫 | 🡫 | 🡫 | 🡫 | 🡫 |
| IFNA21 | 🡫 | 🡫 | 🡫 | 🡫 | 🡫 |
| IFNB1 |  |  |  |  | 🡫 |
| IL1A |  | 🡫 | 🡫 |  | 🡫 |
| Gene | **1mM VPA** | **5mM VPA** | **0.5 µM SAHA** | **2.5 µM SAHA** | **12.5 µM SAHA** |
| IL1F5 |  | 🡫 |  |  |  |
| IL6 |  |  | 🡫 |  |  |
| IL10 | 🡫 | 🡫 |  |  |  |
| IL12B |  | 🡫 |  |  | 🡫 |
| IL23A |  |  |  |  | 🡫 |
| LIF | 🡫 | 🡫 | 🡫 |  | 🡫 |
| LTA |  |  | 🡫 |  | 🡫 |
| OSM |  | 🡫 | 🡫 | 🡫 | 🡫 |
| SCGB3A1 |  |  |  |  | 🡫 |
| TNFSF11 |  | 🡫 |  |  |  |
| TNFRSF11B |  | 🡫 | 🡫 | 🡫 | 🡫 |
| TNFSF15 |  | 🡫 |  |  | 🡫 |
