# Supplementary material for: Cells adapt to the epigenomic disruption caused by histone deacetylase inhibitors through a coordinated, chromatin-mediated transcriptional response
Source: Epigenetics Chromatin. 2015 Sep 16;8:29. doi: 10.1186/s13072-015-0021-9 (PMC4572612; doi:10.1186/s13072-015-0021-9)
Supplement: Additional file 6: — A table showing “pattern specification process genes” that are up-regulated by HDACi treatment. [file 13072_2015_21_MOESM6_ESM.docx]

**Additional Data File 6 - Up-regulation of pattern-specification genes in response to HDACi treatment.**

The table shows genes encoding proteins annotated as involved in “pattern-specification process” that are up-regulated at the inhibitor concentrations shown.

| Gene | 1mM VPA | 5mM VPA | 0.5uM SAHA | 2.5uM SAHA | 12.5uM SAHA |
| --- | --- | --- | --- | --- | --- |
| ACVR2A |  | **↑** | **↑** | **↑** | **↑** |
| ALX4 |  | **↑** |  |  |  |
| ARL6 | **↑** | **↑** | **↑** | **↑** | **↑** |
| ASPH |  | **↑** | **↑** | **↑** | **↑** |
| AXIN2 | **↑** |  | **↑** | **↑** | **↑** |
| BMPR1A |  |  |  | **↑** | **↑** |
| BMPR2 |  | **↑** | **↑** | **↑** | **↑** |
| CTNNBIP1 | **↑** | **↑** |  | **↑** | **↑** |
| CYP26B1 |  | **↑** |  |  |  |
| CYR61 |  | **↑** | **↑** | **↑** | **↑** |
| DLL1 | **↑** | **↑** | **↑** | **↑** | **↑** |
| DLX1 | **↑** |  | **↑** |  | **↑** |
| DLX2 | **↑** |  | **↑** | **↑** | **↑** |
| EFNB1 | **↑** |  |  |  |  |
| FLT1 |  | **↑** |  |  | **↑** |
| FOXJ1 |  | **↑** |  |  | **↑** |
| FRAT1 | **↑** |  | **↑** | **↑** | **↑** |
| GAS1 | **↑** | **↑** | **↑** |  | **↑** |
| GATA4 |  | **↑** |  |  |  |
| GDF11 | **↑** |  |  |  | **↑** |
| GLI1 | **↑** | **↑** | **↑** | **↑** | **↑** |
| HES7 | **↑** |  | **↑** |  | **↑** |
| HHEX | **↑** | **↑** |  | **↑** | **↑** |
| HIPK1 |  | **↑** |  | **↑** | **↑** |
| HOXA1 |  | **↑** | **↑** |  | **↑** |
| HOXA5 |  |  | **↑** |  |  |
| HOXA7 |  |  |  |  | **↑** |
| HOXC6 | **↑** | **↑** | **↑** |  | **↑** |
| KIF3A | **↑** | **↑** | **↑** | **↑** | **↑** |
| Gene | **1mM VPA** | **5mM VPA** | **0.5uM SAHA** | **2.5uM SAHA** | **12.5uM SAHA** |
| KIF3B |  |  |  | **↑** |  |
| LFNG |  |  |  | **↑** | **↑** |
| LHX2 |  |  |  | **↑** |  |
| LRP6 |  | **↑** | **↑** | **↑** | **↑** |
| MIB1 |  |  |  | **↑** |  |
| NEUROD1 | **↑** | **↑** | **↑** | **↑** | **↑** |
| NKX3-1 | **↑** |  |  |  |  |
| NODAL |  |  |  | **↑** | **↑** |
| NR2F2 | **↑** | **↑** | **↑** | **↑** | **↑** |
| PAX6 | **↑** |  |  | **↑** | **↑** |
| PKD2 |  |  |  | **↑** | **↑** |
| RAB23 | **↑** |  |  |  |  |
| SIX1 | **↑** | **↑** | **↑** | **↑** | **↑** |
| SIX3 | **↑** |  |  |  |  |
| SMAD5 |  |  |  | **↑** | **↑** |
| SMAD6 | **↑** | **↑** | **↑** | **↑** | **↑** |
| SMO |  | **↑** |  |  |  |
| TCF7L1 |  |  |  |  | **↑** |
| TGFBR1 |  | **↑** |  |  | **↑** |
| TGFBR2 |  |  |  | **↑** |  |
| TULP3 | **↑** | **↑** |  | **↑** | **↑** |
| WNT1 |  |  |  | **↑** |  |
