# Supplementary material for: Cells adapt to the epigenomic disruption caused by histone deacetylase inhibitors through a coordinated, chromatin-mediated transcriptional response
Source: Epigenetics Chromatin. 2015 Sep 16;8:29. doi: 10.1186/s13072-015-0021-9 (PMC4572612; doi:10.1186/s13072-015-0021-9)
Supplement: Additional file 7: — The effect on the cell cycle and apoptosis of VPA and UNC 1999 treatment. [file 13072_2015_21_MOESM7_ESM.pptx]

## Slide 1
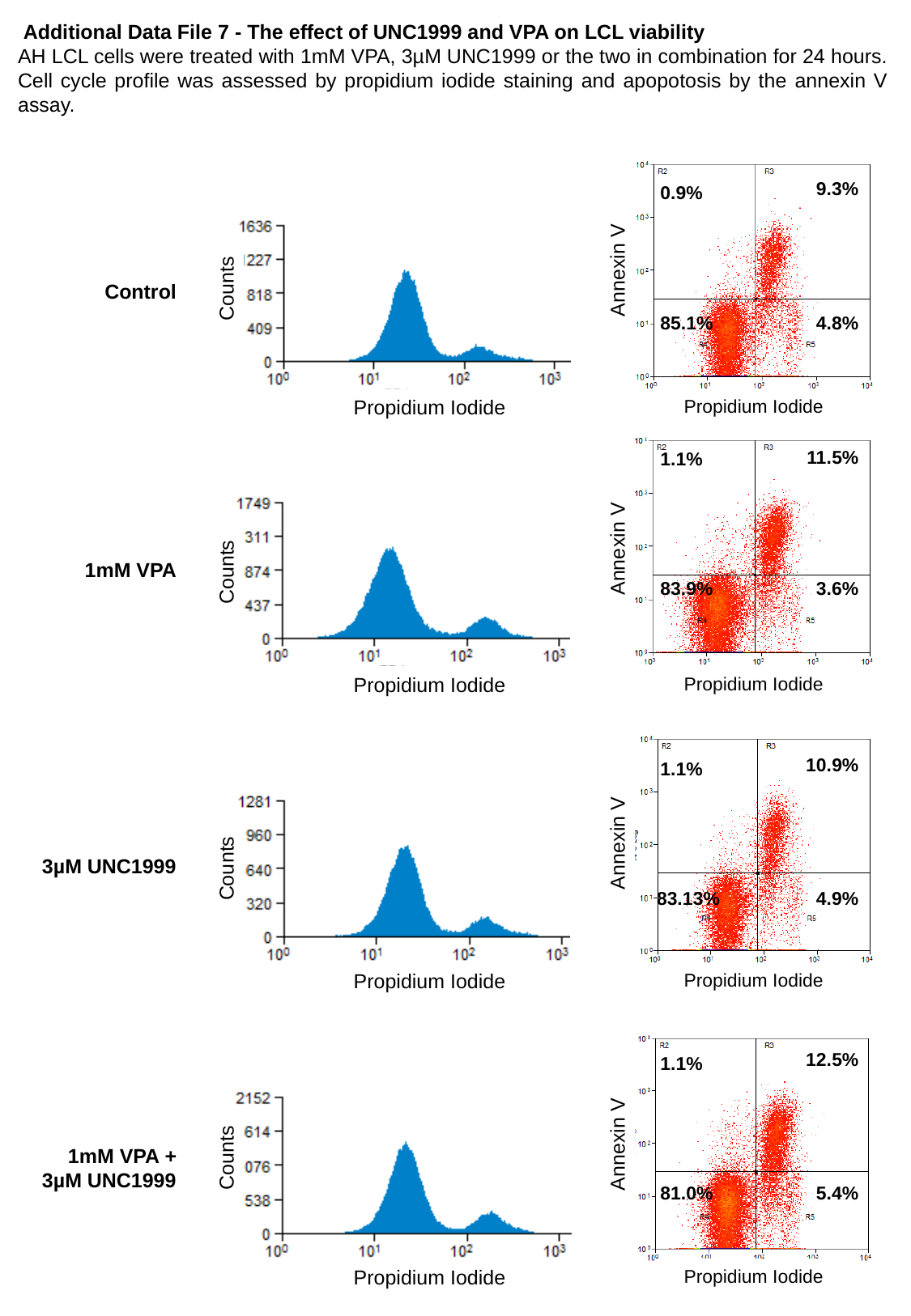

Additional Data File 7 - The effect of UNC1999 and VPA on LCL viability
AH LCL cells were treated with 1mM VPA, 3µM UNC1999 or the two in combination for 24 hours. Cell cycle profile was assessed by propidium iodide staining and apopotosis by the annexin V assay.
9.3%
0.9%
Annexin V
Counts
Control
85.1%
4.8%
Propidium Iodide
Propidium Iodide
11.5%
1.1%
Annexin V
1mM VPA
Counts
83.9%
3.6%
Propidium Iodide
Propidium Iodide
10.9%
1.1%
Annexin V
3µM UNC1999
Counts
83.13%
4.9%
Propidium Iodide
Propidium Iodide
12.5%
1.1%
Annexin V
1mM VPA + 3µM UNC1999
Counts
81.0%
5.4%
Propidium Iodide
Propidium Iodide
