# Supplementary material for: Cells adapt to the epigenomic disruption caused by histone deacetylase inhibitors through a coordinated, chromatin-mediated transcriptional response
Source: Epigenetics Chromatin. 2015 Sep 16;8:29. doi: 10.1186/s13072-015-0021-9 (PMC4572612; doi:10.1186/s13072-015-0021-9)
Supplement: Additional file 8: — Ontology analysis of VPA-responsive genes based on their sensitivity to UNC1999. [file 13072_2015_21_MOESM8_ESM.docx]

**Additional file 8** – Ontology analysis of the effect of EZH1/2 inhibition on VPA response

Human lymphoblastoid cells were treated for two hours with either 1mM VPA, 3µM UNC1999 (an inhibitor of the methyltransferase activity of EZH1/2) or both compounds together. Significant genes were identified by T-Test (P<0.05, fold change >1.5) and sorted by sensitivity to UNC1999 as in figure 7B.

| **Down-regulated genes** | | | | | | |  |
| --- | --- | --- | --- | --- | --- | --- | --- |
| **VPA *not* VPA+UNC1999 (UNC sensitive)** | | | | | | |  |
| Term | Count | | | P-value | | FE |  |
| GOMF: cytokine activity | 9 | | | 8x10^-6^ | | 8.7 |  |
| GOBP: apoptosis | 10 | | | 0.003 | | 3.2 |  |
| GOMF: transition metal ion binding | 25 | | | 0.006 | | 1.7 |  |
|  |  | | |  | |  |  |
| **VPA *and* VPA+ UNC1999 (UNC insensitive)** | | | | | | |  |
| Term | Count | | | P-value | | FE |  |
| GOBP: transcription | 54 | | | 7x10^-13^ | | 2.7 |  |
| GOCC: nuclear lumen | 23 | | | 0.0001 | | 2.4 |  |
| GOCC: histone acetyltransferase complex | 5 | | | 0.0003 | | 14.9 |  |
| GOBP: chemotaxis | 11 | | | 2x10^-6^ | | 7.3 |  |
|  |  | | |  | |  |  |
| **VPA+UNC1999 *not* VPA (UNC dependent)** | | | | | | |  |
| Term | Count | | | P-value | | FE |  |
| GOMF: RNA pol II transcription factor activity | 5 | | | 0.02 | | 5 |  |
| **Up-regulated genes** | | | | | | | |
| **VPA *not* VPA+ UNC1999 (UNC sensitive)** | | | | | | | |
| Term | | Count | P-value | | FE | | |
| GOCC: plasma membrane part | | 44 | 0.002 | | 1.6 | | |
| GOBP: sensory organ development | | 12 | 0.0003 | | 3.8 | | |
| GOBP: tissue morphogenesis | | 9 | 0.003 | | 3.6 | | |
| GOBP: anterior/posterior pattern formation | | 8 | 0.003 | | 4.1 | | |
| GOBP: pattern specification process | | 11 | 0.004 | | 3 | | |
|  | |  |  | |  | | |
| **VPA *and* VPA+ UNC1999 (UNC insensitive)** | | | | | | | |
| Term | | Count | P-value | | FE | | |
| GOMF: transcription factor activity | | 32 | 2x10^-6^ | | 2.5 | | |
| GOBP: pattern specification process | | 14 | 1x10^-4^ | | 3.7 | | |
| GOCC: Golgi apparatus | | 21 | 0.001 | | 2.1 | | |
| GOBP: embryonic organ morphogenesis | | 9 | 6x10^-4^ | | 4.8 | | |
| GOMF: transcription corepressor activity | | 9 | 6x10^-4^ | | 4.8 | | |
|  | |  |  | |  | | |
| **VPA+ UNC1999 *not* VPA (UNC dependent)** | | | | | | | |
| Term | | Count | P-value | | FE | | |
| GOBP: regulation of transcription | | 14 | 0.004 | | 2.2 | | |
| GOMF: transcription repressor activity | | 6 | 0.001 | | 7.7 | | |
| GOCC: membrane raft | | 3 | 0.025 | | 11.7 | | |
